# Social networks and symptomatic and functional outcomes in schizophrenia: a systematic review and meta-analysis

**DOI:** 10.1007/s00127-018-1552-8

**Published:** 2018-06-27

**Authors:** Amy Degnan, Katherine Berry, Daryl Sweet, Kathryn Abel, Nick Crossley, Dawn Edge

**Affiliations:** 1 Division of Psychology and Mental Health, Faculty of Biology, Medicine and Health, The University of Manchester, Zochonis Building; Oxford Road, Manchester, M13 9PL UK; 2 Division of Psychology and Mental Health, The University of Manchester, Manchester, UK; 3 School of Social Sciences, Education and Social Work, Queen’s University, Belfast, UK; 4 School of Social Sciences, The University of Manchester, Manchester, UK

**Keywords:** Psychosis, Schizophrenia, Social networks, Functioning, Symptoms, Quality of life

## Abstract

**Purpose:**

To conduct a systematic review and meta-analysis to examine the strength of associations between social network size and clinical and functional outcomes in schizophrenia.

**Method:**

Studies were identified from a systematic search of electronic databases (EMBASE, Medline, PsycINFO, and Web of Science) from January 1970 to June 2016. Eligible studies included peer-reviewed English language articles that examined associations between a quantitative measure of network size and symptomatic and/or functional outcome in schizophrenia-spectrum diagnoses.

**Results:**

Our search yielded 16 studies with 1,929 participants. Meta-analyses using random effects models to calculate pooled effect sizes (Hedge’s *g*) found that smaller social network size was moderately associated with more severe overall psychiatric symptoms (*N* = 5, *n* = 467, *g* = − 0.53, 95% confidence interval (CI) = − 0.875, − 0.184, *p* = 0.003) and negative symptoms (*N* = 8, *n* = 577, *g* = − 0.75, 95% CI = − 0.997, − 0.512, *p* = 0.000). Statistical heterogeneity was observed *I*^2^ = 63.04%; *I*^2^ = 35.75%,) which could not be explained by low-quality network measures or sample heterogeneity in sensitivity analyses. There was no effect for positive symptoms (*N* = 7, *n* = 405, *g* = − 0.19, 95% CI = 0.494, 0.110, *p* = 0.213) or social functioning (*N* = 3, *n* = 209, *g* = 0.36, 95% CI = − 0.078, 0.801, *p* = 0.107). Narrative synthesis suggested that larger network size was associated with improved global functioning, but findings for affective symptoms and quality of life were mixed.

**Conclusion:**

Psychosocial interventions which support individuals to build and maintain social networks may improve outcomes in schizophrenia. The review findings are cross-sectional and thus causal direction cannot be inferred. Further research is required to examine temporal associations between network characteristics and outcomes in schizophrenia and to test theoretical models relating to explanatory or mediating mechanisms.

**Electronic supplementary material:**

The online version of this article (10.1007/s00127-018-1552-8) contains supplementary material, which is available to authorized users.

## Introduction

Social connections can have positive effects on mental health, for example, by directly increasing self-esteem or buffering the negative effects of socioenvironmental stressors [1, 2]. Having a greater number of friends has been associated with lower depressive symptomatology [3, 4], which may be explained by higher social integration and improved sense of belonging [3]. Conversely, having fewer social connections is associated with adverse outcomes, such as poorer health and increased risk of early mortality [5]. However, social relationships are not always supportive and can be sources of conflict and stress [6]. For example, emotionally over-involvement and hostile interactions with significant others can lead to higher rates of relapse in individuals diagnosed with schizophrenia [7, 8]. Social withdrawal may be used as a protective mechanism, but this can further limit the availability of social connections and important buffers, thereby increasing the risk of poor outcomes [9, 10].

Over the past few decades, an abundance of research has shown that social networks are disrupted in individuals diagnosed with schizophrenia and psychosis. Social networks can be described as the set of social relations or social ties that connect individuals [11]. Commonly used measures of social networks in the mental health literature include network size (i.e., the number of persons), frequency of contact, and the quality of relationships between individuals (e.g., social support, satisfaction, and emotional closeness). Research has consistently reported smaller and poorer quality networks in people with severe mental health problems when compared to the general population [12, 13]. It is often assumed that the size and quality of social networks diminish as a consequence of psychosis, with earlier theories proposing a ‘network crisis’ at first onset [14, 15]. This has been contradicted by findings that network characteristics are relatively stable over the year following initial hospitalisation [16]. Recent evidence suggests that social networks and satisfaction with social support deteriorate at first episode and before the onset of psychosis [9]. It is now generally accepted that the relationship between network disruption and increasing chronicity is non-linear with network changes occurring prior to and at the later stages of psychosis [9].

Social network is a multidimensional construct, yet research in schizophrenia and psychosis tends to use generic measures and focuses on functional attributes such as social support [17]. Social network analysis (SNA) [18, 19] provides a comprehensive method of describing and analysing social networks, defined as sets of social ties or connections between individuals. SNA draws a distinction between structural characteristics, or the patterns of social connections, and interactional characteristics, such as the content, function or quality of relationships. This approach minimises bias as it delineates the effects of objective characteristics of social relationships from individual-level subjective variables [16]. Structural features of social networks that have received the most attention in schizophrenia research are size, composition, and density (i.e., interconnectedness). Compared to non-psychotic populations, the social networks of people diagnosed with schizophrenia and psychosis tend to be smaller and more interconnected, comprising proportionately more family members and fewer friends [20, 21]. However, social network characteristics have been shown to vary substantially across individuals and samples [21, 22], with research, suggesting that these differences may be associated with outcomes in schizophrenia [16, 23]. In addition, to objective symptomatic and functional outcomes, studies have examined subjective outcomes such as perceived quality of life (QOL) [24].

Despite the potential importance of network characteristics for outcomes in schizophrenia, to date, there has been no systematic review of the magnitude or nature of these relationships. The previous literature reviews on networks and outcomes are outdated, not systematic, include mixed diagnostic samples, and do not focus specifically on network size and service user-related outcomes [25, 26]. These reviews also fail to differentiate structural from interactional network characteristics (in their relationship with outcomes) and do not conduct formal quality assessments considering the heterogeneous measurement of social networks in relation  to study findings [27]. This paper addresses this gap in the literature by providing a systematic review and meta-analysis of studies examining the relationship between the social network size and outcomes in schizophrenia. The present review focused on network size as this has been the most commonly cited measure of social networks in the literature, with relatively few studies conducted on other structural characteristics.

The specific aims of this review were to: (1) carry out a systematic search and narrative synthesis on the nature and strength of the relationship between social network size and symptomatic, functional and QOL outcomes in schizophrenia; (2) examine the quality of the empirical findings and the measurement of social networks; and (3) conduct a series of meta-analyses to examine the magnitude of the relationship between network size and schizophrenia outcomes. The findings will determine whether social networks are important for outcomes and highlight potential targets for psychosocial interventions.

## Method

The review was conducted in accordance with Preferred Reporting Items for Systematic Reviews and Meta-analyses (PRISMA) guidelines [28]. The review protocol was registered on PROSPERO [29].

### Eligibility criteria

Eligible studies were peer-reviewed journal articles published in English. Studies published after 1970 were included as these were the first empirical studies of social networks in schizophrenia [30, 31]. Included studies comprised a sample of participants who were at least 18 years of age and majority (≥ 70%) schizophrenia spectrum diagnosis based on: (1) ICD (ICD-9 or -10 F20-29) or DSM criteria (i.e., schizophrenia, schizoaffective disorder, delusional disorder, schizophreniform disorder, or psychosis not otherwise specified) or (2) clinical evaluation of non-affective psychosis in Early Intervention Services. Articles were quantitative empirical studies examining associations between social networks and current symptomatic, functional or QOL outcomes in schizophrenia. Eligible designs included cross-sectional and longitudinal studies, with no restriction on the direction of the relationship. However, retrospective measures of premorbid symptoms or functioning were excluded. Studies were required to include at least one quantitative measure of social network size and current symptomatic, functional, or QOL outcome in schizophrenia.

### Search strategy

On 1 June 2016, a systematic electronic search was conducted on EMBASE, Medline, PsycINFO and Web of Science. Several combinations of the following and related search words were used and separated by the Boolean operators OR and AND: ‘schizophrenia’ OR ‘psychosis’ OR ‘severe mental illness’ AND ‘social network’ OR ‘personal network’ OR ‘social tie’. Medical Subject Headings (MeSH) and explode functions were used to expand the search and identify all relevant studies. Given that we were investigating multiple outcomes, we did not include outcome-related search terms to ensure we covered all literature. The search strategy was adapted for each database (supplementary **S1**).

### Screening and study selection

Two authors (AD and DS) independently screened articles for eligibility. Titles and abstracts were examined against the inclusion and exclusion criteria (stage 1). Full texts of potentially relevant articles were retrieved and screened and those that met the inclusion criteria were retained (stage 2). Level of agreement at stage 1 was 90% and stage 2 was 89%. At each stage of screening, discrepancies were resolved via discussion with KB before continuing to the next stage. Additional studies were identified through scanning reference lists of included articles.

### Narrative synthesis

A narrative synthesis [32] was carried out to summarise and critically appraise the reviewed studies. Empirical findings were combined into a narrative by categorising outcomes into coherent theoretical domains. Effect sizes were presented in tables where available.

### Quality assessment

The Effective Public Health Practice Project (EPHPP) Quality Assessment Tool for Quantitative Studies [33] was used to evaluate study quality. The EPHPP has been applied to healthcare-related systematic reviews with demonstrable inter-rater reliability and content and construct validity [34, 35]. We adapted the tool to be consistent with the observational analytic design of the included studies. Components relating to randomised designs, blinding, and intervention integrity were omitted. Given the heterogeneity of social network measures in schizophrenia research [27], it was important to include a separate assessment of their quality. The EPHPP in the current review, therefore, included six components: (1) selection bias; (2) confounders; (3) data collection–outcome; (4) data collection–social network; (5) withdrawals and drop-outs; and (6) analysis. Each component was rated as either ‘strong’, ‘moderate’ or weak’. The lead author (AD) and a postgraduate student conducted the quality assessments. Substantial agreement was found (*k* = 0.610–0.888). Discrepancies were discussed and resolved with KB.

### Meta-analysis

#### Eligibility criteria

Studies that statistically examined associations between social network size and a validated outcome measure were included in the meta-analyses. Studies were excluded if there was insufficient data to calculate effect sizes, despite attempts to contact authors for missing data.

#### Data extraction and effect size computation

Data were available for separate meta-analyses on the relationship between network size and (1) overall psychiatric symptoms; (2) positive symptoms; (3) negative symptoms; and (4) social functioning. Most studies reported cross-sectional correlational analyses (Pearson’s *r* or Spearman’s rho) which were converted to the common metric Hedge’s *g* for meta-analysis. For studies reporting regression, the effect size *r* was estimated and converted to Hedge’s *g*.

Consistent with previous meta-analyses in the field [36, 37], we developed a protocol to minimise the potential effects of non-independent data, improve comparability across studies and reduce bias: (1) where studies reported cross-sectional and temporal associations, cross-sectional data were used; (2) when longitudinal studies reported cross-sectional results at multiple timepoints, data from the earliest timepoint were used (Time 1/baseline); and (3) where studies reported multivariate analyses and adjusted for covariates, the unadjusted data were used.

### Statistical analysis

Comprehensive Meta-analysis version 3.0 [38] was used to calculate effect sizes and perform meta-analyses. Random effects models were used due to considerable variation across study measures assessments and designs. The model performs better than fixed-effect models and provides more conservative estimates accounting for observed heterogeneity [39, 40]. Heterogeneity was examined using Cochran’s Q and *I*^2^ statistics to determine the amount of heterogeneity resulting from variance between studies (*p* < 0.05). Visual inspection of funnel plots and Egger’s test of funnel plot asymmetry was applied to examine publication or selection bias. For meta-analyses demonstrating significant effects, the Fail-Safe N was calculated to estimate the number of additional unpublished/missing studies that would be required to nullify the effect.

Sensitivity analyses were conducted removing studies with weak or moderate quality network measures (as indicated from the quality assessment) and samples with < 100% schizophrenia/non-affective psychosis. ‘One-study-removed’ analyses were conducted to assess whether any studies skewed the results.

## Results

### Study selection

**Fig. 1:**
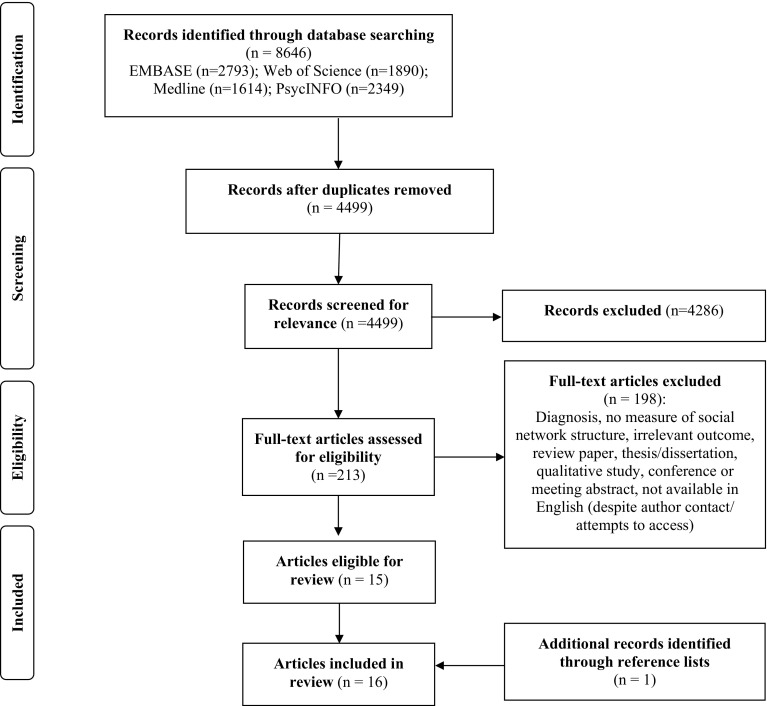
PRISMA diagram

The search across all databases yielded 15 articles for inclusion. One additional article was identified through searching reference lists resulting in a total of 16 articles. The study selection process is summarised in the PRISMA diagram (Fig. 1).

### Study characteristics

Four of the 16 included articles used overlapping samples. Two studies [41, 42] comprised the same sample as two earlier studies [43, 44], but addressed different aims. Of the 14 independent samples, five were conducted in the USA, four in the UK, two in Poland, and one in each of Australia, Denmark, and Austria. There were a total of 1929 independent participants across the included studies at baseline, with sample sizes ranging from 24 to 547. Of these, 1102 (57%) were male. Of 11 studies reporting it, the mean age ranged from 23 to 63 years. Seven of 12 studies reporting ethnicity were mostly (> 60%) Caucasian. Seven studies included multiple ethnic groups (UK—Black-Caribbean, Black-African, Asian; USA—Latino and African American). Ten of the 14 independent samples were 100% schizophrenia spectrum; including four first episode and seven schizophrenia diagnoses. Two samples were mixed (affective and non-affective) psychosis and two were severe mental illness (SMI) that also included affective disorders. Seven studies reported mean duration of illness which ranged from 2.3 to 16.7 years. Most studies were cross-sectional, with only three longitudinal studies examining temporal associations [45–47]; all of which were randomised controlled trials (RCTs). Study characteristics and key findings are presented in Table 1. Sample characteristics can be found in supplementary (S2).

**Table 1:** Characteristics and key findings of included studies

Author, year, country	Sample* N*(M/F) % SS	Social network measure	Outcome measure	Outcome category	Design (timepoints)	Key findings
**Allison et al. (2013)** ***UK***	24 (18/6) 100%	Modified PRQ	PANSS (P, N) HADS	Symptom severity (positive, negative & affective)	Cross-sectional correlational	1. No sig correlations between size of network and positive (rho = − 0.15), negative (rho = − 0.27), anxiety (rho = − 0.09), or depression (rho = 0.01) symptoms
**Angell & Test (1992)** ***USA***	87 (62/25) 98%	CAF—study specific	BPRS-18 (P)	Symptom severity (positive)	Cross-sectional correlational + longitudinal (18 + 24 months)	1. N = 122 in RCT, but complete data for 87 (71.3%) due to attrition 2. Cross-sectional analyses at T1 showed no sig correlation between positive symptoms and network size (rho = − 0.07) 3. Cross-sectional analyses at T2 showed no sig correlation between positive symptoms and network size (rho = − 0.18) 4. OLS regression showed no sig association between T1 positive symptoms and T2 network size (*β* = − 0.11) over six month period, controlling for education, gender, age and treatment allocation 5. OLS regression found increase in positive symptoms over a six month period (T1 to T2) was sig associated with declines in network reciprocity (*β* = − 0.23) but not size (*β* = − 0.17)
Becker et al. (1998) *UK*	143 (80/63) 83%	SNS	LQoLP	QOL	Cross-sectional correlational (baseline)	1. Sample grouped according to quintiles of network size, from 1 (small network, 1–6 contacts) to 5 (large network, 19–42 contacts) 2. ANOVA showed sig quadric contrast between social network quintiles in terms of average QOL. Average QOL increased up to quintiles 4 and 5; sig contrasts between these two and the lower three quintiles 3. In multiple linear regression analyses, average QOL was sig positively associated with higher network quintiles, with levelling off at around 20 contacts (*β* = 0.61, CI = 0.25, 0.96). This remained after adjusting for age, BPRS anxiety/depression, service satisfaction, and number of unmet needs
**Cechnicki & Wojciechowska (2008)** ^**1**^ ***Poland***	64 (28/36) 100%	BQ	BPRS-24 (G, N, P) DSM-III: social functioning	Symptom severity (overall, positive, negative) Social functioning	Cross-sectional correlational (7 years after first admission)	1. Larger social networks sig associated with less severe BPRS overall (rho = − 0.32), positive (rho = − 0.42) and negative symptoms (rho = − 0.38) and improved social functioning (rho = 0.38) 2. Large extra-familial networks sig related to less severe BPRS overall (rho = − 0.29), negative (rho = − 0.30) and positive (rho = − 0.38) symptoms and improved social functioning (rho = 0.44)
Cechnicki et al. (2008)^1^ *Poland*	64 (28/36) 100%	BQ	LQoLQ	QOL	Cross-sectional correlational (7 years after first admission)	1. Large social network sig correlated with higher general subjective satisfaction with QOL (rho = 0.35) 2. Larger extra-familial network did not correlate with general QOL (rho = 0.12)
Cohen et al. (1997) *USA*	117 (29/88) 100%	Modified NAP	TSC	QOL	Cross-sectional correlational	1. 47% (*n* = 54) satisfied and 53% (*n* = 63) not satisfied with their lives and QOL over the past 30 years 2. Satisfied group sig more likely to have more network members who could be ‘counted on’ (*t* = − 2.52, *d* = − 0.47, CI = − 0.84, 0.10) and greater network density (*t* = − 2.73, *d* = − 0.51, CI = − 0.88, − 0.14) 3. No sig group differences in total network size (*t* = 0.90, *d* = 0.17, CI = − 0.20, 0.53) 4. Multivariate logistic regression model showed more reliable social contacts (could be ‘counted on’) sig predicted greater satisfaction with QOL, adjusting for socio-demographics (AOR = 4.68, CI = 1.36, 16.08). Network density was not a sig predictor of QOL (AOR = 2.94, CI = 0.43, 20.18)
**Cresswell et al. (1992)** ***UK***	40 (31/9) 100%	SNIS	BPRS-18 (G) SANS	Symptom severity (overall and negative)	Cross-sectional correlational	1. Higher severity of overall symptoms sig related to smaller primary group (family/friends) (*r* = − 0.36), secondary group (outside family/friends) (*r* = − 0.47) and primary group seen weekly (*r* = − 0.32) 2. Higher severity of negative symptoms sig related to smaller network size in primary group (*r* = − 0.42) and primary group seen weekly (*r* = − 0.41), but not secondary group (*r* = − 0.21)
**Dixon et al. (2001)** ^**2**^ ***USA***	218(123/95) 72%	Study specific	PANSS (T)	Symptom severity (overall)	Cross-sectional, correlational	1. Hierarchical OLS regression showed that total PANSS symptom severity was inversely associated with the size of social support network (*r* = − 0.21), which remained when controlling for demographic covariates (age, education, gender, ethnicity) (*sr*^*2*^ = 0.041)
**Goldberg et al. (2003)** ^**2**^ ***USA***	218(123/95) 72%	Modified SSSNI	PANSS (GP, P, N) BQOL	Symptom severity (affective, positive, negative) QOL	Cross-sectional, correlational	1. Smaller network size was sig correlated with more severe negative symptoms (*r* = − 0.29) and general symptoms (*r* = − 0.19). Positive symptoms and general satisfaction with QOL were not sig associated with network size (na) 2. Results from ANOVA showed sig differences across five network density categories for PANSS general (*F* = 3.30), positive (*F* = 3.14) and negative (*F* = 3.00) symptoms, with trend for less severe symptoms for low to moderate density and more severe symptoms for no connections. But post-hoc tests only sig for general symptoms. QOL was not sig associated with density (na)
**Hamilton et al. (1989)** ***USA***	39 (39/0) 100%	Modified PPKI	NSRS and SANS SAPS	Symptom severity (positive and negative)	Cross-sectional, correlational	1. NSRS total scores sig negative correlated with social network size (rho = − 0.64) 2. Positive symptoms on SANS did not correlate significantly with network size (na)
**Horan et al. (2006)** ***USA***	T1: 89 (75/14) T2: 34(na) 100%	Study specific	BPRS-24 (G, P, N, A) SAS	Symptom severity (overall, positive, negative, affective) Social functioning	Cross-sectional correlational (baseline, 15 months)	1. At baseline (T1), total network size did not sig correlate with BPRS overall (*r* = 0.01), BPRS positive (*r* = 0.11), BPRS negative (*r* = − 0.16), BPRS anxiety/depression (*r* = 0.07) or current overall social functioning (total SAS) (rho = 0.03). Higher percent kin was sig associated with lower total SAS scores (*r* = − 0.28). Network density and degree did not correlate with BPRS symptoms or functioning 2. 34/87 participants (38%) completed T2 assessments. At 15 months, smaller total network size correlated with more sig severe BPRS positive symptoms (*r* = − 0.36), but did not sig correlate with BPRS overall (*r* = − 0.19), BPRS anxiety/depression (*r* = 0.05) or BPRS negative (*r* = − 0.16). Higher scores on BPRS positive correlated with greater density (*r* = 0.42) and lower per cent kin (*r* = − 0.37), but not degree. BPRS negative and overall symptoms did not correlate with density, percent kin or degree at T2 3. Current social functioning did not correlate with size (*r* = 0.19), density (*r* = 0.05), degree (*r* = 0.05) or percent kin (*r* = − 0.05) at T2
Howard et al. (2000) *UK*	302 (143/159) 74%	SNS	GAF (D)	Global functioning	Cross-sectional correlational + longitudinal (baseline, 2 years)	1. Complete data *n* = 135 Time 1 and *n* = 130 Time 2. 188 (62%) completed SNS T1 and 151 (50%) T2; 230 (76%) completed GAF T1 and 215 (71%) T2. Reasons not reported 2. Cross-sectional regressions found positive associations between social network size and GAF disability at T1 (*n* = 135, β = 0.22, CI = 3.46. 11.62) and T2 (*n* = 130, *β* = 0.19, CI = 0.73, 12.89) 4. No sig temporal association between social networks T1 and GAF disability T2 controlling for GAF at T1 (*n* = 107, *β* = 0.14, CI = − 0.35, 12.36) 5. The best fitting model from SEM (*n* = 107) suggested that total social network size at T1 explains some variance in GAF disability at T1 (coefficient = 0.19), and GAF T1 explains some of the variance in GAF over a 2 years period (T2) (coefficient = 0.34), but insufficient power to detect effects
**Macdonald et al. (1998)** ***Australia***	46 (34/12) 100%	SRS—two subscales	SAPS SANS BDI	Symptom severity (positive, negative, affective)	Cross-sectional correlational	1. No sig correlations between network variables and depressive (na) or positive symptoms (*r* = 0.07). Size of support network sig correlated with increased negative symptoms (*r* = 0.34) 2. SEM supported a tentative model that negative symptoms has a direct effect (coefficient = − 0.31) on social skill, with an indirect effect (coefficient = 0.34) on the size of social networks via social skill. This model accounted for 15% of the variance in the size of networks
Sibitz et al. (2011) *Austria*	157 (85/72) 100%	Study specific	WHOQOL-BREF ADS	QOL Symptom severity (affective)	Cross-sectional, correlational	1. Increased number of friends sig correlated with lower depressive symptoms (*r* = − 0.32) and improved subjective QOL (*r* = 0.27) 2. SEM showed that poorer social network (insufficient number friends) negatively influences subjective QOL only if leading to stigma and low empowerment, which resulted in depression and, in turn, impaired QOL (indirect effect = 0.16). There was no direct effect of QOL (direct effect = − 0.07) on social network
Thorup et al. (2006) *Denmark*	547(323/224) 100%	SNS	SAPS SANS GAF (S, F)	Symptom severity (positive, negative) Global functioning	Cross-sectional correlational and longitudinal (baseline, 2 after treatment onset)	1. 369/547 (67%) completed entire 2 year follow-up. After missing data and attrition, Time 1 *N* = 505, Time 2 *N* = 347 2. Reduction in negative symptoms and global functioning sig associated with larger network size (family and friends) at baseline and 2 years 3. Reduction in disorganised symptoms associated with larger network size at 2 years but not baseline 4. Positive symptoms was not sig related to network size at baseline or 2 years 5. Multivariate regression models at 2 years (*N* = 332) included site, treatment, age and number of contacts at entry as sig covariates. Male gender (− 0.86), older age (− 0.07) and more severe disorganised symptoms (− 0.29) were sig predictors of reduced family network size. More severe negative symptoms (− 0.26), older age (− 0.04) and completed A-level status (0.74) were sig predictors of reduced friendship network size
**Wojciechow et al. (2002)** ***Poland***	56 (32/24) 100%	BQ	BPRS-24 (G, N, P) DSM-III: social functioning	Symptom severity (overall, positive, negative) Social functioning	Cross-sectional correlational (3 years after first admission)	1. Smaller total network size sig correlated with greater intensity of BPRS overall (rho= -0.49), negative (rho= -0.48) and positive (rho= -0.29) symptoms. No sig correlations between size of network and social functioning (rho = 0.14) 2. Larger extra-familial network sig correlated with fewer overall (rho = − 0.42) and positive symptoms (rho = − 0.30) and improved social functioning (rho = 0.55). No sig correlations between extra-familial network and negative symptoms (rho = − 0.25)

*Social network measures BQ* Bizon’s Questionnaire (Bizon et al. 2001); *CAF* Community Adjustment Form (Test et al. 1991); *NAP* Network Analysis Profile (Cohen and Sokolovsky 1979, 1981); *PPKI* Pattison Psychosocial Kinship Inventory (Pattison et al. 1981); *PRQ* Peer Relations Questionnaire (Connolly and Johnson 1996); *SNIS* Social Network Interview Schedule (Sheperd 1984); *SNS* Social Network Schedule (Dunn et al. 1990); *SSSNI* Social Support and Social Network Interview (Lovell et al. 1984); *SRS* Social Relationships Scale (McFarlane et al. 1981)

*Outcome measures ADS* Allgemeine Depressions Skala (Hautzinger and Bailer 1993), German version of Centre for Epidemiological Studies Depression Scale (CES-D) (Radloff 1997); *BDI* Beck’s Depression Inventory (Beck and Steer 1987); *BPRS-24* Brief Psychiatric Rating Scale (24 items) (Overall and Gorham 1962); BPRS-18 (Overall and Klett 1972); BPRS-P = positive subscale (Mueser et al. 1997b); *BPRS G* global scores; *N* negative subscale; *BQOL* Brief Quality of Life Inventory (Lehman et al. 1995); *DSM III* Diagnostic and Statistical Manual of Mental Disorders third edition (American Psychiatric Association 1980); *GAF* Global Assessment of Functioning (Endicott et al. 1976); *GAF-D* GAF disability subscale; *HADS* Hospital Anxiety and Depression Scale (Zigmond and Snaith 1983); *LQoLQ* Lehman’s Quality of Life Questionnaire (1988); *LQoLP* Lancashire Quality of Life Profile (Oliver 1991; Oliver et al. 1996); *NSRS* Negative Symptom Rating Scale (Iager et al. 1985); *PANSS* Positive and Negative Syndrome Scale (Kay et al. 1987); *PANSS T* total scores; *N* negative, *P* positive, *GP* general psychopathology subscales; *SAS* Social Attainment Scale (Goldstein 1978); *SANS* Scale for the Assessment of Negative Symptoms (Andreason 1984); *SAPS* Scale for the Assessment of Positive Symptoms (Andreasen 1982); *SF-36* The 36 item Short Form Survey (Ware and Sherbourne 1992; Ware et al. 1993); *TSC* The SHORT CARE (Gurland et al. 1984); *WHOQOL-BREF* World Health Organisation-Quality of Life Assessment-26 item version (WHOQOL Group 1998)

*ANOVA* Analysis of variance, *AOR* adjusted odds ratio, *CI* 95% confidence intervals, *F* female, *M* male, *na* not reported or not available, *OLS* ordinary least squares regression, *QOL* quality of life, *RCT* randomised controlled trial, *SS* schizophrenia spectrum, *SEM* structural equation modelling, *sig* statistically significant, *T1* Time 1, *T2* Time 2

^1,2^Studies used the same samples; studies highlighted in bold text are in the meta-analysis

### Social network characteristics

A broad range of different measures were used to assess social networks. Assessment tools included structured or unstructured interviews, questionnaires, single-item measures, and rating scales. Network definition and criteria varied in terms of the time period or amount of social contact (e.g., present month, past month, and contact every month) and the number of network members, with some studies setting a limit on the number of people named (e.g., maximum of 10) and others asking for a list of all people known. Mean total network size was reported for six independent samples and ranged from 4.18 [41] to 12.9 [48]. Characteristics of the social network measures are available in supplementary (S3).

### Study quality assessment

Quality assessments are presented in Table 2. Selection bias was rated weak for 59% (*n* = 16) of studies due to lack of detail on recruitment and selection procedures, self-referred or convenience sample or less than 60% response rate. Eighteen studies controlled for confounders in the analyses or design (*n* = 5 rated ‘moderate’ as 1 + confounders, and *n* = 12 ‘strong’ as 2 + confounders). Data collection for outcomes was rated ‘strong’ for just over half (*n* = 14) studies reporting valid and reliable outcome measures. The remaining studies were given ‘moderate’ (*n* = 5) and ‘weak’ (*n* = 8) ratings mainly because of poor reporting of service use data collection (e.g., hospital admissions) and no references for translated measures which brought ratings down (despite studies including validated measures for other outcomes). Fifty-nine percent (*n* = 16) of social network tools were rated as strong. Network tools were rated as ‘weak’ in seven studies due to non-validated assessment tools with inadequate measure of network size; including lack of detail (*n* = 2), boundaried (capped network size or focus on one type of relation) (*n* = 3), single-item measures (*n* = 2), and no measure of size (*n* = 1). ‘Moderate’ ratings were given to four studies (11%) due to lack of detail (*n* = 2) or boundaried networks (*n* = 2). Withdrawals and drop-outs was rated ‘not applicable’ for the vast majority of studies (*n* = 23) due to the cross-sectional design and rated ‘moderate’ for two longitudinal studies with 60–79% follow-up rate and weak for two studies with less than 60% follow-up rate. Most analysis sections (*n* = 24) were appropriate to the research aims and statistical methods appropriate for the design and were marked as ‘strong’ (n = *9*) or ‘moderate’ (*n* = 15). Fifteen studies were marked as ‘moderate’ for analyses due insufficient detail relating to the management of missing data, distribution and skewness, power analyses, and correction for multiple correlations.

**Table 2:** Methodological quality of included studies

Study reference	Selection bias	Confounders	Data collection—outcome	Data collection—size	Withdrawals and drop-outs	Analyses
Allison et al. (2013)	WEAK	WEAK	STRONG	MOD	N/A	MOD
Angell and Test (1992)	WEAK	STRONG	STRONG	WEAK	MOD	MOD
Becker et al. (1998)	MOD	STRONG	STRONG	STRONG	N/A	STRONG
Cechnicki & Wojciechowska (2008)^1^	WEAK	WEAK	STRONG	STRONG	N/A	MOD
Cechnicki et al. (2008)^1^	WEAK	WEAK	WEAK	STRONG	N/A	MOD
Cohen et al (1997)	MOD	STRONG	STRONG	STRONG	N/A	STRONG
Cresswell et al. (1992)	WEAK	WEAK	STRONG	STRONG	N/A	WEAK
Dixon et al. (2001)^2^	WEAK	STRONG	STRONG	WEAK	N/A	STRONG
Goldberg et al. (2003)^2^	MOD	WEAK	STRONG	WEAK	N/A	STRONG
Hamilton, et al. (1989)	WEAK	WEAK	STRONG	STRONG	N/A	MOD
Horan et al. (2006)	WEAK	WEAK	STRONG	STRONG	WEAK	MOD
Howard, Leese & Thornicroft (2000)	MOD	STRONG	STRONG	STRONG	WEAK	STRONG
Macdonald et al. (1998)	WEAK	STRONG	STRONG	MOD	N/A	STRONG
Sibitz et al. (2011)	MOD	STRONG	STRONG	WEAK	N/A	STRONG
Thorup et al. (2006)	WEAK	MOD	STRONG	MOD	MOD	STRONG
Wojciechow et al. (2002)	WEAK	WEAK	STRONG	STRONG	N/A	MOD

*MOD* moderate, *N/A* not applicable

^1,2^Overlapping samples

### Association between social networks and outcomes

**Fig. 2:**
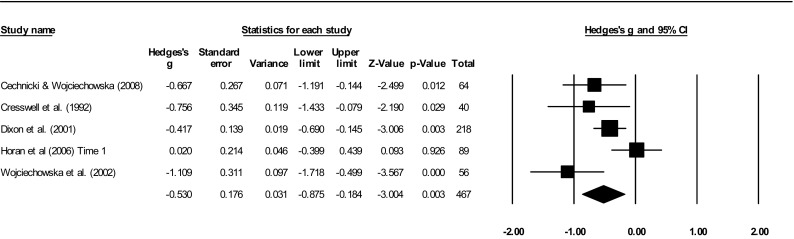
Forest plot for total symptoms

A total of 12 studies were included in the meta-analyses on the association between social network size and outcomes. Two studies [41, 43] had overlapping samples, but measured different outcomes and were included in separate analyses. See Table 3 for summary statistics and Fig. 2 for forest plot for overall psychiatric symptoms. See supplementary for forest plots (S4) for other outcomes and funnel plots (S5).

**Table 3:** Summary statistics for meta-analyses and sensitivity analyses: social network size and outcomes in schizophrenia

Outcome	Studies	Total *N*	Random effects meta-analysis				Heterogeneity		
			Hedge’s *g*	95% CI		*p* value	*Q* value *(df)*	*p* value	*I*^2^ (%)
Overall symptoms									
Total	5	467	− 0.530	− 0.875	− 0.184	**0.003**	10.822 (4)	0.029	63.037
100% SS + high-quality network	4	249	− 0.595	− 1.111	− 0.079	**0.024**	10.683 (3)	0.014	71.919
Positive symptoms									
Total	7	405	− 0.192	0.494	0.110	0.213	12.709 (6)	0.048	52.788
100% SS	6	318	− 0.206	− 0.581	0.169	0.281	12.683 (5)	0.027	60.578
High-quality network	4	248	− 0.276	− 0.793	0.241	0.296	11.357 (3)	0.010	73.584
Negative symptoms									
Total	8	577	− 0.754	− 0.997	− 0.512	**0.000**	10.895 (7)	0.143	35.748
100% SS	7	358	− 0.818	− 1.126	− 0.509	**0.000**	10.128 (6)	0.119	40.757
High-quality network	5	288	− 0.899	− 1.319	− 0.480	**0.000**	9.789 (4)	0.044	59.138
Social functioning									
Total	3	209	0.361	− 0.078	0.801	0.107	4.737 (2)	0.094	52.766

Bold figures indicate statistically significant association between social network size and outcome

*SS* Schizophrenia spectrum, *CI* confidence interval

#### Symptomatic outcomes

##### Overall psychiatric symptoms

Meta-analyses of five studies with 467 participants showed a significant moderate effect (*g* = − 0.53) for the association between smaller network size and overall psychiatric symptoms, with moderate heterogeneity (*I*^2^ = 63.04%). Egger’s regression test was non-significant (*t* = 1.06, SE = 2.16, *p* = 0.365), indicating no publication or selection bias (Fail-Safe *N* = 28). A sensitivity analysis removing one study [43] with < 100% schizophrenia sample and a poor quality network measure slightly increased the effect (*g* = − 0.60) and heterogeneity (*I*^2^ = 71.92%).

The significant results are based on cross-sectional evidence from four studies of samples with longer term problems [43, 44, 49, 50]. One recent onset study [16] found no cross-sectional associations between size and symptoms at initial hospitalisation or 15 months. However, the disruptions in network characteristics typical of schizophrenia samples (i.e., small, densely interconnected, and high proportion kin) were present at initial hospitalisation and remained stable at follow-up. Although this study included strong quality network and outcome measures, it did not examine the association longitudinally or control for potential confounders. Dixon et al. [43] were the only study to consider the influence of extraneous variables and showed that symptoms contributed to reduced network size when entered into a regression model with demographic covariates (i.e., age, gender, education, and ethnicity). However, this study included affective diagnoses and used a poor quality network measure (i.e., single item).

##### Positive symptoms

Seven studies with 405 participants were included in the meta-analysis for positive symptoms which found no significant effect of network size (*g* = − 0.19) and moderate heterogeneity (*I*^2^ = 52.79%). Egger’s test indicated no publication bias (*t* = 0.56, SE = 2.82, *p* = 0.598). A sensitivity analysis excluding one study [45] with < 100% schizophrenia made little difference to the findings (*g* = − 0.21; *I*^2^ = 60.58%). Removing three studies with weak [45] and moderate [23, 51] quality network measures also had a negligible effect (*g* = − 0.28, *I*^2^ = 73.58%).

Findings for positive symptoms were mixed. Three studies found a significant cross-sectional association between larger size and less severe positive symptoms [16, 42, 50], but this was not supported in six studies [23, 41, 45, 47, 48, 51]. Two RCTs [41, 47] with non-significant findings were omitted from the meta-analysis due to insufficient data. One of these [41] was the only study that did not suffer from selection bias, though it was also the only study to include a mixed diagnostic sample which may have affected its external validity.

The other omitted study [47] was one of the two RCTs in first episode psychosis to report longitudinal analyses and adjust for confounders. Thorup et al. [47] found that more severe disorganised symptoms were related to a reduction in family but not friendship network size over a two year period, adjusting for treatment allocation, age, and social network size at baseline. There was no association between positive symptoms and number of social contacts. Angel and Test [45] showed that an increase in positive symptoms over a  six month period was not related to network size, controlling for education, gender, and treatment allocation. Their study had a small sample and may have lacked sufficient power to detect effects. In addition, the measure of network size only included non-kin and was capped at ten members. Both longitudinal studies were rated as moderate quality for withdrawals and drop-outs based on the fact that around 70% of the sample were analysed at follow-up after attrition and missing data. Both studies reported no significant differences between those who were and were not followed up in terms of demographic, network, and outcome measures at baseline. However, the selection procedures in both studies were rated low quality as the response rate was not reported.

##### Negative symptoms

Meta-analysis conducted on eight studies (*n* = 577) showed a significant negative association between network size and negative symptoms (*g* = − 0.75) and low heterogeneity (*I*^2^ = 35.75%). There was no evidence of publication bias as indicated by Egger’s test (*t* = 1.75, SE = 1.04, *p* = 0.131; Fail-Safe *N* = 123). A sensitivity analysis removing one study [41] with less than 100% schizophrenia found a slight increase in effect (*g* = − 0.82) and heterogeneity (*I*^2^ = 40.76%). An additional sensitivity analysis removing three studies [23, 41, 51] with low-quality network measures also increased the effect size (*g* = − 0.90) and heterogeneity (*I*^2^ = 59.14%).

Seven studies [41, 44, 47–51] reported a significant association. Two first episode psychosis studies did not find an association; Allison et al. [23] comprised a small sample with poor quality network measure (i.e., capped at ten), but the study by Horan et al. [16] was higher quality. All but one study [41] rated low quality on selection bias, as a result of convenience sampling or lack of detail on recruitment and selection procedures.

Only two studies adjusted for confounders, one of which examined the relationship longitudinally. In their first episode sample, Thorup et al. [47] suggested that more severe negative symptoms predicted reduced friendship but not family network size over a  two year period, adjusting for treatment allocation, age, and number of contacts at baseline. Macdonald et al. [51] explored the influence of social skill in the relationship between negative symptoms and total network size in schizophrenia using structural equation modelling. Cross-sectional analyses supported a tentative model to suggest that negative symptoms have an indirect effect on the size of social networks via social skill, accounting for 15% of the variance in the model.

##### Affective symptoms

Five cross-sectional studies examined affective symptoms. Having fewer friends was weakly related to more severe depressive symptoms in a large schizophrenia sample [52]. In a large SMI sample [41], smaller total network size weakly correlated with more severe general psychopathology. However, findings were not consistently supported. Friendship size did not relate to depression or anxiety in a small first episode sample [23]. Total network size did not correlate with depression in a study of schizophrenia outpatients [51] or with depression or anxiety in first episode psychosis [16]. Each study used a different outcome measure, though all were validated. Both studies with significant findings [41, 52] were stronger quality in that they had larger samples and lower selection bias compared to the other studies. However, they did not control for confounders and were cross-sectional. Moreover, the network measures were of low quality (i.e., capped at ten [41] and single items [52]).

#### Functional outcomes

##### Social functioning

Three studies (*n* = 209) measured social functioning outcomes. Meta-analyses showed no significant effect (*g* = 0.36) and moderate heterogeneity (*I*^2^ = 57.77%). Egger’s test was non-significant (*t* = 1.22, SE = 6.67, *p* = 0.437), suggesting no selection bias. All studies had 100% schizophrenia samples and high-quality social network measures. Sensitivity analyses indicated that the removal of one study [44] resulted in a substantial reduction in effect size (*g* = 0.14) and heterogeneity (*I*^2^ = 0%). This study assessed outpatients seven years after the initial hospitalisation, whereas the other two included patients in earlier stages of schizophrenia. No studies adjusted for confounders.

##### Global functioning

Two longitudinal RCTs [46, 47] reported cross-sectional associations between more social contacts and improved global functioning, but no significant temporal relationship. Howard et al. [46] referred to weak evidence from structural equations to suggest that networks could affect functioning over a two year period. However, the study reportedly lacked power to detect effects. Although the analyses controlled for age and ethnicity, they included a mixed diagnostic sample with patients at different stages of illness, but did not adjust for diagnosis or illness duration. Thorup et al. [47] adjusted for confounders (treatment group, age, and number of contacts) and included a number of covariates in multivariate analyses, but global functioning did not predict family or friendship network size over two years. Both had considerable attrition at follow-up (33 and 40%, respectively), with weak evidence that participants who dropped out were those who had greater difficulties, and thus, the generalisability of these results is questionable.

##### Quality of life

Five cross-sectional studies examined QOL outcomes [24, 41, 42, 52, 53]. Higher subjective QOL was associated with having more social contacts in two schizophrenia samples [42, 52]. In one of these, further analyses using structural equation modelling found no direct effect of social network size on QOL. However, a tentative model showed a small indirect effect of reduced number of friends on QOL through higher perceived stigma and low empowerment, which led to depression and subsequently impaired QOL. This study comprised a large sample of long-term schizophrenia patients, but did not adjust for variation in symptom severity and included a poor quality network measure (i.e., single item).

One high-quality study in a large random sample of psychosis [24] found that satisfaction with average QOL was positively associated with larger social networks, with a tailoring off at around 20 social contacts. Multivariate analyses showed that age, anxiety and depression, service satisfaction, and needs for care were also independently associated with QOL, but did not confound its association with network size.

However, findings were mixed and two studies in patients with longer term problems found no relationship between number of social contacts and QOL [41, 53]. One of these [41] was rated poor quality as it did not control for confounders and included a poor quality measure of network size (i.e., capped at ten). The other study [53] was of strong quality and included a relatively large sample, but the participants were over 55 years of age and thus unlikely representative of younger people at earlier stages of psychosis.

## Discussion

This is the first systematic review and meta-analysis on the relationship between social network size and outcomes in schizophrenia. Meta-analytic pooled effect sizes found that smaller social network size was moderately associated with more severe overall psychiatric symptoms and negative symptoms, but not positive symptoms or social functioning. There was low statistical heterogeneity between studies for negative symptoms and moderate heterogeneity for overall psychiatric symptoms. Our narrative review highlighted some evidence to show that a having more social ties is moderately associated with better global functioning, fewer affective symptoms and improved satisfaction with QOL.

Two of the 16 studies in this review examined potential mechanisms to explain the processes by which a greater number of social ties is associated with improvements in negative symptoms and QOL in schizophrenia; via social skill [51], and stigma and empowerment [52], respectively. However, most of the reviewed studies reported cross-sectional data, and thus, causal direction cannot be inferred. Larger social networks may lead to improved symptoms by buffering stress associated with schizophrenia, but negative symptoms such as anhedonia and apathy may also impede individuals’ motivation and social skills and reduce their tendency to build relationships [47]. Only three studies examined temporal associations and, taken together, suggest a bi-directional relationship; with significant results showing that more severe disorganised symptoms predict smaller networks [47] and smaller networks predict poorer global functioning [46]. It is likely that the relationship is reciprocal and that there is a complex interplay between more disrupted social networks, individual characteristics, such as social skill, stigma and empowerment, and poorer outcomes over time.

There was limited evidence that the relationship between network size and outcomes may be non-linear. Findings from one study indicated a curvilinear relationship to suggest that service users with around 20 network members experience a better QOL [24]. This suggests that there may be an optimum network size for improved outcome. Larger network structures may allow more resources such as information and support but can also be overwhelming, stressful and come with certain expectations or constraints. Moderately sized networks, with a sufficient number of social contacts, may be more manageable while still enabling access to sufficient resources for coping [26, 54].

There are some methodological issues to consider when interpreting the findings. Methodological quality can influence effect sizes [55]. Sensitivity analyses were conducted, therefore, removing studies with low-quality social network measures and less than 100% schizophrenia spectrum samples; these slightly increased the effect size for overall psychiatric and negative symptoms. However, removal of these studies also increased statistical heterogeneity, suggesting that there were other unmeasured sample or study characteristics that accounted for heterogeneity. In addition to diverse network measures, our quality assessment highlighted variation in methodology such as selection procedures and study design which may have affected the results. One limitation is that we were unable to explore potential moderator effects in the meta-analyses due to the small number of studies and insufficient data. Our meta-analyses included cross-sectional univariate data and, therefore, can only tell us about association.

It is plausible that some of the reviewed studies did not find an association, because they did not consider other unmeasured variables that may be related to network structure or outcome. Only half of the reviewed studies controlled for confounding effects of clinical and socio-demographic variables in multivariate analyses (e.g., symptoms, age, ethnicity, and gender). Based on current evidence, it is difficult to determine the effects of social network characteristics and outcomes independent of confounders or other explanatory or mediating mechanisms [6, 10]. More sophisticated statistical analyses in larger samples are required to test theoretical models which identify potential mediators, effect moderators, and causal pathways. Future controlled trials of interventions that measure changes to networks alongside changes to clinical and functional outcomes, at multiple timepoints, would allow better inference about causation and the direction of the effect.

There was a tendency for network size to be more strongly related to symptomatic and functional outcomes in individuals at later stages of schizophrenia when compared to first episode. This was supported by evidence for stronger associations the longer the time period from previous hospitalisation [16, 42, 47]. Experiencing a psychotic episode and a period of hospitalisation for the first time is likely to be very stressful and chaotic; during this time, it is plausible that people are less able to access or mobilise resources within their social networks to help manage symptoms or engage in social activity [16]. No studies controlled for illness duration and few controlled for diagnosis. Future research would benefit from adjusting for and drawing comparisons between subgroups within the schizophrenia spectrum and at different stages of illness.

Social networks were measured using a variety of assessment tools based on different definitions, timescales, and criteria, as previously highlighted in psychosis research [9, 27, 56]. It is often assumed that having more network members is beneficial as this corresponds to greater levels of support [9]. However, social connections may be appraised negatively and consist of over involved, unhelpful, or critical interactions. Other features of the network are also likely to interact with the structure of the network to influence outcome, such as the function, content, and perceived quality of social ties. Focusing on network size may not be the primary goal and it is important to reflect on person-centred formulations to consider what meaningful and resourceful social contact is for the individual [22]. It would be fruitful for future research and clinical practice to use comprehensive network-mapping assessment tools that examine the different types of relationships, transactional qualities (e.g., reciprocity, frequency, and intensity), and structure of social networks (e.g., density).

To conclude, our findings indicate that larger social networks are associated with better symptomatic and functional outcome in schizophrenia. Interventions that target social networks may, therefore, indirectly improve these outcomes. Controlled trials using longitudinal designs are required to confirm whether supporting an individual to increase the number of people in their social networks leads to a reduction in symptoms. Given that network changes can occur prior to and during the early stages of schizophrenia [9], clinicians should intervene early to support individuals to access and mobilise their social connections during a period of stability after initial contact with services [16]. Psychosocial interventions such as peer support, community engagement, and social skills training can lead to improvements in the size of social networks in psychosis [56]. Clinical guidelines for the management of schizophrenia and psychosis recommend peer support and self-management interventions for building social support networks [57]. These should focus on skills to develop and maintain social connections in diverse and important areas in the person’s life, including family, friends, and professional relations. Network enhancement interventions may include strategies that target stigma and empowerment [52], and social skills [51], though these hypothesised mechanisms require further investigation. Supporting individuals to map out their social connections in diagrammatic form would be helpful to provide a better understanding of social networks from their perspective [22, 58]. A network mapping approach may be useful in understanding how different network characteristics might beneficial at different stages of recovery [59]. Finally, the rapid adoption and endorsement of mobile technologies in mental health research [60] may present a novel, cost-effective, and feasible way for accurately measuring and building social networks in schizophrenia and psychosis. Analysis of such data would provide information relating to how social network characteristics and interactions may differ between individuals and how this relates to symptomatic and functional outcomes. These findings suggest a role for routine use of network mapping tools which could also be used therapeutically to inform more person-centred clinical practice as well as to measure networks as predictors and outcomes in clinical trials.

## Electronic supplementary material

Below is the link to the electronic supplementary material.

Supplementary material 1 (DOCX 93 KB)
