# Supplementary material for: Social networks and symptomatic and functional outcomes in schizophrenia: a systematic review and meta-analysis
Source: Soc Psychiatry Psychiatr Epidemiol. 2018 Jun 27;53(9):873–88. doi: 10.1007/s00127-018-1552-8 (PMC6133157; doi:10.1007/s00127-018-1552-8)
Supplement: Supplementary file 1 — Supplementary material 1 (DOCX 93 KB) [file 127_2018_1552_MOESM1_ESM.docx]

**SOCIAL NETWORKS AND OUTCOMES IN SCHIZOPHRENIA**

**SUPPLEMENTARY MATERIAL**

S1: Search strategy

S2: Sample characteristics of included studies

S3: Characteristics of social network measures

S4: Forest plots

S5: Funnel plots

**SUPPLEMENTARY 1**

**SEARCH STRATEGY**

**EMBASE [1974 to 2016 June 01]**

**=2793**

1. exp schizophrenia/
2. exp psychosis/
3. (psychotic or schizo$ or psychosis or psychoses). ti.sh. hw.ab.kw.
4. [title, subject headings, heading words, abstract, key word]
5. ((chronic$ or sever$) adj5 mental$ adj5 (ill$ or disorder$)).ti.hw.ab.kw.sh
6. or/1-4
7. exp social network/
8. (network$) adj5 (social or famil$ or peer or friend$).ti.sh. hw.ab.kw.
9. (Egonet$ or ‘personal net$’ or ‘egocentric net$’). ti.sh. hw.ab.kw.
10. (tie$ or relation$ or contact$ or connect$) adj5 (social). ti.sh. hw.ab.kw.
11. or/ 6-9
12. 5 and 10

**PsycINFO** [**1806 to May Week 4 2016]**

**=2349**

1. exp schizophrenia/
2. exp psychosis/
3. (psychotic or schizo* or psychosis or psychoses).ab.hw.id.sh.ti [abstract, heading word, key concepts, subject heading, title]
4. ((chronic* or sever*) adj5 mental* adj5 (ill* or disorder*)). ab.hw.id.sh.ti
5. or/1-4
6. exp social networks/
7. (network*) adj5 (social or famil* or peer or friend*). ab.hw.id.sh.ti
8. (Egonet* or “personal net*” or “egocentric net*”). ab.hw.id.sh.ti
9. (tie* or relation* or contact* or connect*) adj5 (social). ab.hw.id.sh.ti
10. or/6-9
11. 5 and 10
12. Limit 11 to yr=’1970-current’

**MEDLINE [1946 to May Week 4 2016]**

**=1614**

1. exp schizophrenia/
2. exp psychotic disorders/
3. (psychotic or schizo* or psychosis or psychoses).ab.hw.kf.kw.sh.ti [abstract, subject heading word, keyword heading word, keyword heading, MeSH subject heading, title]
4. ((chronic* or sever*) adj5 mental* adj5 (ill* or disorder*)).ab.hw.kf.kw.sh.ti
5. or/1-4
6. (network*) adj5 (social or famil* or peer or friend*). ab.hw.kf.kw.sh.ti
7. (Egonet* or “personal net*” or “egocentric net*”).ab.hw.kf.kw.sh.ti
8. (tie* or relation* or contact* or connect*) adj5 (social). ab.hw.kf.kw.sh.ti
9. or/6-8
10. 5 and 9
11. Limit 11 to yr=’1970-current’

**WEB OF SCIENCE**

**Timespan: 1970-2016. Indexes: SCI-EXPANDED, SSCI.**

**=1890**

1. TS=(psychotic OR schizo* OR psychos$s) [= TOPIC: title, abstract, author key words, key word plus]
2. TS=((chronic* OR sever*) NEAR/5 mental* NEAR/5 (ill* OR disorder*))
3. OR/1-2
4. TS=((network*) NEAR/5 (social OR famil* OR peer OR friend*))
5. TS=(egonet* OR “personal net*” OR ‘egocentric net*’)
6. TS=((tie* OR relation* OR contact* OR connect*) NEAR/5 (social))
7. OR/4-5
8. 3 AND 7

**SUPPLEMENTARY 2**

**S2: Sample characteristics of included studies**

| **Author, year, country** | **Setting/ sample** | **Diagnosis**  **(% SS; screening tool)** | **Duration SS, years (Mean)** | **Total N (M/F)** | **Age, years (Mean^4^)** | **Ethnicity (N/%)** |
| --- | --- | --- | --- | --- | --- | --- |
| Allison et al. (2013)  *UK* | EIP teams | 100%  ICD-10 F20-29 clinical referral | 2.3^3^ | 24 (18/6) | 23 | WB (11/46%), Black (2/8%), Asian (6/25%), Other (4/17%) |
| Angell & Test (1992)  *USA* | Secondary analysis data PACT trial: RCT of assertive community treatment in young adults with early onset schizophrenia | 98% (2% schizotypy)  clinical referral | na | 87 (62/25) | N/%  20-24 (44, 50)  25-29 (26, 30)  30-32 (17, 20) | Caucasian (83/95%), AA (3/4%), Latino (1/1%) |
| Becker et al. (1998)  *UK* | Prospective controlled evaluation study of community mental health services in two inner city areas – baseline data for random sample of psychosis | 83% (17% AD)  OPCRIT functional psychosis; ICD-10 SCAN | 16.2 | 143 (80/63) | 38.6 | White (87/61%), BC (41/29%), BA (11/8%) |
| Cechnicki & Wojciechowska (2008) **^1^**  *Poland* | Krakow longitudinal study: community outpatient ward for psychosis rehab – evaluated 7 years after first hospitalisation | 100%  DSM-III clinical diagnosis | na | 64 (28/36) | 32 | Na |
| Cohen et al. (1997)  *USA* | Older adults (≥55 years) with schizophrenia living in community | 100%  DSM III-R clinical diagnosis | na | 117 (29/88) | 63 | White (41/35%), Black (47/39%), Latino (29/25%) |
| Cresswell et al. (1992)  *UK* | Day outpatients at rehab centre inner-city hospital | 100%  Hospital case notes, clinical report | 16.7 | 40 (31/9) | 41.7 | Caucasian (26/65%); non-Caucasian (14/35%) |
| Dixon et al. (2001)^2^  *USA* | Reanalysis data from EIDP: multi-site RCT comparing two vocational interventions for people with SMI | 72% (38% AD)  DSM-IV SCID | na | 218(123/95) | 41 | Caucasian (53/24%), AA (143/65%), Other (11/23%) |
| Hamilton et al. (1989)  *USA* | Veterans mental health clinic (>2 years schizophrenia) | 100%  DSM-III clinical diagnosis, research screening interview | 11 | 39 (39/0) | 33 | Caucasian (33/85%) |
| Horan et al. (2006)  *USA* | Subsample DPSD project: longitudinal study schizophrenia with first episode psychosis (<2 years) | 100%  DSM-III-R, PSE | 1.3 | T1: 89 (75/14)  T2: 34(na) | T1: 23.8  T2: na | Caucasian (77/86%) |
| Howard et al. (2000)  *UK* | PRiSM psychosis study; prospective non-randomised controlled trial two community mental health services | 74% (17% AP, 9% OP)  OPCRIT functional psychosis; ICD-10 SCAN | na | 302 (143/159) | Med=40  Range=15-89 | Caucasian (193/64%), BC (74/24%), BA (17/6%), Other (14/5%), missing (4/1%) |
| Macdonald et al. (1998)  *Australia* | Outpatients three inner-suburban community mental health clinics (≥ 2 months discharged acute episode) | 100%  DSM-III-R; psychiatrist report & interview clinical psychologist | 5.6 | 46 (34/12) | 34(9.3) | Na |
| Sibitz et al. (2011)  *Austria* | Inpatients & outpatients of medical university & various mental health centres | 100%  ICD-10 criteria | 13.5 | 157 (85/72) | 37.3 | White (100%) |
| Thorup et al. (2006)  *Denmark* | Subsample OPUS trial; RCT integrated versus usual treatment in first episode psychosis - inpatient & outpatient services | 100%  ICD-10 SCAN | Na | 547(323/224) | Range=18-45 | Na |
| Wojciechow et al. (2002)  *Poland* | Krakow longitudinal study: community outpatient ward for psychosis rehab – evaluated 3 years after first hospitalisation | 100%  DSM-III clinical diagnosis | na | 56 (32/24) | 28 | Na |

*Note:*

Studies deleted as same study samples: ^1^ Cechnicki et al. (2008), Poland; ^2^Goldberg et al. (2003), USA; ^3^defined as first contact with services; ^4^mean unless otherwise stated

*Trials/studies*

DPSD=Developmental Processes in Schizophrenia Disorders project (Nuechterlein et al., 1992); EIDP=Employment Intervention Demonstration Program (Lehman et al., 2002); LPD=The Study of Low Prevalence disorders (Jablensky et al., 2000); PACT= Programme of Assertive Community Treatment (Test et al., 1991); PRiSM psychosis study (Thornicroft et al., 1998); OPUS trial (Peterson et al., 2005).

*Diagnostic screening measures*

DSM=Diagnostic and Statistical Manual of Mental Disorders (American Psychiatric Association: DSM-III,1980; DSM-III-R, 1987; DSM-IV, 1994); ICD-10= International Classification of Disease-10 (WHO, 1992); ICD-9 (WHO, 1978); ICD F20-29= schizophrenia, schizoaffective disorder, delusional disorder, schizophreniform disorder, or psychosis not otherwise specified; LIS=Lifetime Illness Review (Jeste, 1997); PSE=Present State Examination (Wing et al., 1974); OPCRIT=Operational Criteria Checklist (McGuffin et al., 1991); SCAN= Schedules for Clinical Assessment in Neuropsychiatry (World Health Organisation, 1992ab); SCID (First et al., 1994).

*Abbreviations*

AD=affective disorders; AA=African American; AP=affective psychosis; BA=Black African; BC=Black Caribbean; EIP=Early Intervention in Psychosis; F=female; M=male; Med=median; na=not reported or not available; OP=other psychosis; RCT=randomised controlled trial; SMI=serious mental illness; SS=schizophrenia spectrum; T1=Time 1; T2=Time 2; UK; United Kingdom; USA=United States of America; WB=White British.

**SUPPLEMENTARY 3**

**S3: Characteristics of social network measures**

| **Author, year, country** | **Measure** | **Assessment tool** | **Network criteria** | **Time period** | **Network members /composition*** | **Network size**  **Time 1**  **M (SD)^** | **Network size**  **Time 2**  **M (SD)^** | **Network variables** |
| --- | --- | --- | --- | --- | --- | --- | --- | --- |
| Allison et al. (2013)  *UK* | Modified PRQ | Questionnaire | Maximum of 10 friends, including same sex best friend | Present | Friends | 4.3 (2.91) | - | **Size** |
| Angell & Test (1992)  *USA* | CAF - study specific | Semi-structured interview | Maximum of 10 non-kin/ non-professional close friends | Past month | Friends | T1: 2.5 (2.94) | T2: 2.7 (2.82) | **Size**  Reciprocity  Intensity of opposite sex contact  Satisfaction with social relationships  Loneliness |
| Becker et al. (1998)  *UK* | SNS | Semi-structured interview | Number of network contacts (analysed in quintiles from 1 (small) to 5 (large)) | Past month | *Total*  Friends  Relatives  Non-friends | 12.8  4.1  4.1  3.9 | - | **Size**  Composition  Number of confidants  Intensity of interaction: number of active, intermediate and passive contacts |
| Cechnicki & Wojciechowska (2008) **^1^**  *Poland* | BQ | Questionnaire | Number of persons currently in contact with (≤10=small; 10>20=average; >21=large) | Present | Total ≤10  Total 10>20  Total >21  Extra-familial | 24%  45%  31%  na | - | **Size**  **Composition**  Age of network  Type of support system  Amount of support  Localisation of support |
| Cohen et al. (1997)  *USA* | Modified NAP | Semi-structured interview | Number of people with notable interaction (e.g. 15 min conversation, material exchange or social outing; includes informal & formal) | Past 3 months for non-kin and 12 months for kin | Total | na  na  na | - | **Size**  **Density**  Proportion of intimates  Proportion of linkages can count on  Proportion of sustenance relations |
| Cresswell et al. (1992)  *UK* | SNIS | Semi-structured interview | Primary (family and friends) and secondary (professionals, neighbours, local shop workers, workmates, service users, acquaintances) network members | Seen or in contact past 12 months and seen weekly | Primary 12 months  Primary weekly  Secondary weekly | 7  32  3 | - | **Size**  **Composition** |
| Dixon et al. (2001)^2^  *USA* | Study specific | Single question | Number of people who meet instrumental and/or affiliative needs | Present | Support network | na | - | **Size** |
| Goldberg et al. (2003)^2^  *USA* | Modified SSSNI | Semi-structured interview | Maximum ten critical members of social network who provide support in some capacity | Present | *Total*  MH friends  Non-MH friends Friends (both) Relatives  MH professionals | 4.18(2.27)  M% (SD)  12.1(23.6)  30.8(31.1)  42.9(30.6)  37.4(31.7)  19.6(25.2) | - | **Size**  **Density**  Composition  Multiplexity |
| Hamilton et al. (1989)  *USA* | Modified PPKI | Semi-structured interview (+ verified list via significant other) | Number of kin and non-kin | Present | *Total*  Kin | 12.9(9.2)  6.2(5.1) | - | **Size**  Composition  Number of multiplex relations (kin and non-kin)  Instrumental relations (kin and non-kin)  Reciprocal relations (kin and non-kin)  Contact frequency (high, low, moderate for kin and non-kin) |
| Horan et al. (2006)  *USA* | Study specific | Semi-structured interview | Number of people in contact with and/or feels close to | 12 month period prior to hospitalisation | *Total*  Nuclear family  Extended family  Share residence  Percent kin | T1: 8.8(3.8)  T1: 3.9(2.4)  T1: 1.7(2.1)  T1: 1.8(1.4)  T1: 0.6(0.30) | T2: 8.7(3.0)  T2: 3.3(1.5)  T2: 1.1(1.8)  T2: 2.2(1.5)  T2: 0.5(0.2) | **Size**  **Composition**  **Density**  **Degree**  Reciprocity (receives more, equal, provides more, reciprocity score) |
| Howard et al. (2000)  *UK* | SNS | Semi-structured interview | Number of network contacts | Past month | Total  Friends  Relatives  Non-friends | Med(R)  10(1-56)  3(0-35)  3(0-17)  3(0-30) | Med(R)  15(2-43)  4(0-35)  4(0-26)  4(0-28) | **Size**  Composition  Number of confidants  Intensity of interaction: number of active, intermediate and passive contacts |
| Macdonald et al. (1998)  *Australia* | SRS – two subscales | Rating scale – Likert response | Maximum of six people whom person talks to for six different areas of life (work, money and finances, home and family, personal and social, personal health, societal issues) | Present | *Total* | 6.3(3) | - | **Size**  Average amount of perceived social support |
| Sibitz et al. (2011)  *Austria* | Study specific | Single question | Number of social contacts with four possible answers (‘no or little social contacts’, ‘few acquaintances’, ‘few close friends’, ‘sufficient friends and acquaintances’) | Present | Number of friends  No or little social contacts  Few acquaintances Few close friends  Sufficient friends and acquaintances | 4.6(5.0)  13(8.3)  16(10.2)  51(32.5)  77(49) | - | **Size / composition** |
| Thorup et al. (2006)  *Denmark* | SNS | Semi-structured interview | Number of family and friends in contact with (up to maximum of 25) | Previous month | *Total* | T1=7.6 | T2=8.2 | **Size**  **Composition** |
| Wojciechow et al. (2002)  *Poland* | BQ | Questionnaire | Number of persons currently in contact with: ≤10=small; 11>20=average; >21=large | Present | Total  Extra-familial | na  na | - | **Size**  **Composition**  Age of network  Type of support system  Amount of support  Localisation of support |

*Note:*

Description of measures based on information reported in the articles;***only some network types mutually exclusive*;* **^1^**Cechnicki et al. (2008) deleted as reports same network characteristics for same study sample; ^2^report on overlapping samples but different network characteristics; ^ M and SD, unless otherwise stated; bold text is structural characteristics that have been examined in relation to outcomes in the studies (some studies described structural characteristics but not did not examine their association with outcomes).

*Abbreviations*

M=mean; Med=median; MH=mental health; na=not reported or not available; R=range; SD=standard deviation; T1=Time 1; T2=Time 2.

*Social network measures*

BQ=Bizon’s Questionnaire (Bizon et al., 2001); CAF=Community Adjustment Form (Test et al., 1991); NAP=Network Analysis Profile (Cohen & Sokolovsky, 1979, 1981); PPKI=Pattison Psychosocial Kinship Inventory (Pattison et al., 1981); PRQ=Peer Relations Questionnaire (Connolly & Johnson, 1996); SNIS=Social Network Interview Schedule (Sheperd, 1984); SNS=Social Network Schedule (Dunn et al., 1990); SSSNI=Social Support and Social Network Interview (Lovell et al., 1984); SRS=Social Relationships Scale (McFarlane et al., 1981).

**SUPPLEMENTARY 4**

**FOREST PLOTS**

**S4.1 Forest plot of the association between social network size and negative symptoms**

**S4.2 Forest plot of the association between social network size and positive symptoms**

**S4.3 Forest plot of the association between social network size and social functioning**

**SUPPLEMENTARY 5**

**FUNNEL PLOTS**

**S5.1 Funnel plot for the association between social network size and overall psychiatric symptoms**

**S5.2 Funnel plot for the association between social network size and negative symptoms**

**S5.3 Funnel plot for the association between social network size and positive symptoms**

**S5.4 Funnel plot for the association between social network size and social functioning**
